# Impact of an Innovative Classroom-Based Lecture Series on Residents' Evaluations of an Anesthesiology Rotation

**DOI:** 10.1155/2016/8543809

**Published:** 2016-02-17

**Authors:** Pedro Tanaka, David Yanez, Hendrikus Lemmens, Adam Djurdjulov, Lena Scotto, Lindsay Borg, Kim Walker, Sylvia Bereknyei Merrell, Alex Macario

**Affiliations:** ^1^Department of Anesthesiology, Perioperative and Pain Medicine, 300 Pasteur Drive H3580, Stanford University School of Medicine, Stanford, CA 94305-5640, USA; ^2^Biostatistics University of Washington, Seattle, WA 98195, USA; ^3^Stanford University School of Medicine, Stanford, CA 94305-5640, USA; ^4^Hoag Memorial Hospital Presbyterian, Newport Beach, CA 92663, USA

## Abstract

*Introduction*. Millennial resident learners may benefit from innovative instructional methods. The goal of this study is to assess the impact of a new daily, 15 minutes on one anesthesia keyword, lecture series given by faculty member each weekday on resident postrotation evaluation scores.* Methods*. A quasi-experimental study design was implemented with the residents' rotation evaluations for the 24-month period ending by 7/30/2013 before the new lecture series was implemented which was compared to the 14-month period after the lecture series began on 8/1/2013. The primary endpoint was “overall teaching quality of this rotation.” We also collected survey data from residents at clinical rotations at two other different institutions during the same two evaluation periods that did not have the education intervention.* Results*. One hundred and thirty-one residents were eligible to participate in the study. Completed surveys ranged from 77 to 87% for the eight-question evaluation instrument. On a 5-point Likert-type scale the mean score on “overall teaching quality of this rotation” increased significantly from 3.9 (SD 0.8) to 4.2 (SD 0.7) after addition of the lecture series, whereas the scores decreased slightly at the comparison sites.* Conclusion*. Rotation evaluation scores for overall teaching quality improved with implementation of a new structured slide daily lectures series.

## 1. Introduction

In 2012 as part of the Annual Program Evaluation for our anesthesiology residency (24 anesthesia residents/year), the department prioritized improving the evaluations by the house staff of the multispecialty anesthesia rotation. In this clinical rotation, residents work in the operating room (OR) providing care to adults having a variety of orthopedic, abdominal, urology, and ambulatory surgeries. Eight to twelve residents per month are on this rotation making it the largest in the training program.

Prior to the current study, several teaching interventions were investigated to improve the “education” of the rotation as the house staff residents reported that there was “not enough teaching.” The first attempted intervention was to add an online daily learning module based on an American Board of Anesthesiology (ABA) keyword that the attending and resident could review on the OR computer during the day. Keywords are two or three word items describing some aspect of anesthesia knowledge to be tested on the In-Training Exams that anesthesia residents take every year. A second intervention was to employ an early morning didactic series of lectures before the start of the workday. Neither of these interventions was adopted. The primary reasons were that there was a perceived lack of consistent time for one-on-one teaching in the OR and that there was not a database with a fully detailed content for each and all the ABA keywords.

Focus groups with the house staff were then conducted to better identify possible interventions that would improve residents' overall learning experience and perceptions of teaching quality. The discussions revealed that house staff wanted focused and explicit instruction of medical knowledge. The recommended delivery of this information was for a lecture series intervention. We devised short, 15-minute time limited lectures that would be given by the same faculty member, repeated three times per day. The objectives were to (a) increase access for all residents to be able to attend the lectures, given the constraints of their individual schedules, and (b) minimize extraneous faculty-to-faculty variation in the information provided to the residents for a particular topic. Additionally, a predetermined template developed by a group of residents was provided to the faculty lecturers for their presentation slides to provide additional uniformity for how a given lecture's content would be delivered. This template included having a brief clinical case scenario to introduce the day's ABA keyword topic and a multiple choice written board style question to promote interaction between the lecturer and residents.

This resulting educational intervention has been fully adopted every weekday of the year and continues almost 24 months after initial implementation. We believed this intervention of a daily 15-minute slide based lecture series on one ABA keyword given by the same faculty member each weekday at 10AM, noon, and 2PM daily would improve the rotation evaluations. The goal of this study is to assess residents' postrotation evaluations before and after the education intervention and specifically the primary endpoint, mean scores on the “overall teaching quality of this rotation.” The average scores of the seven other postrotation survey questions were also studied.

## 2. Methods

This study was approved by the Stanford University Institutional Review Board as an exempt study. The faculty in the Department of Anesthesiology, Perioperative and Pain Medicine, selected topics for the intervention lectures from the keyword list available at http://www.openanesthesia.org/category/aba-keywords/.

The choice of keywords was not directly related to anesthesia cases in the surgical suite that day. All 32 members of the department's attendings that work in the multispecialty division and supervise residents during the anesthesia rotation participated as faculty in the lecture series and used the same standardized slide template developed by a group of residents and faculty with expertise in medical education. The 10-slide maximum template included a case vignette, background information, summary of important information, review of original case, and a written multiple choice question [1] and summary of key take home points.

A quasi-experimental study design was implemented to compare the residents' rotation evaluations. We considered the time period from 7/1/2011 to 7/30/2013 (24 months before new lecture series) to serve as the “control” time, and the time period from 8/1/2013 to 10/1/2014 (14 months after lecture series implemented) to serve as the intervention period. We wished to evaluate whether there were differences in the study endpoints between the two periods. All residents in the program were invited to participate in the evaluations over the 38 monthly time periods.

The primary endpoint was for the survey question “overall teaching quality of this rotation.” This question and the six other survey questions are displayed as follows:

1. the goals of the rotation defined;
2. the goals of the rotation achieved;
3. quality of the syllabus;
4. the cases of educational value;
5. clinical teaching;
6. teaching not directly involved with case management;
7. feedback provided;
8. overall teaching quality of this rotation.

Each question was rated by the residents using a 5-point Likert-type scale: 1: Unsatisfactory, 2: Below Average, 3: At Expectation, 4: Above Expectation, and 5: Outstanding. Data were collected online via the MedHub medical education management software (MedHub, Inc., Ann Arbor, Michigan) used by all residencies at our institution. The residents' scores were not identifiable to specific residents; the residents were unaware of this study.

The lecture series “intervention” took place at Stanford University Medical Center. Another rotation at the VA Palo Alto Health Care System (VA) and one at the Santa Clara County Valley Medical Center (Valley) did not have an education intervention. We collected data from our residents at these “control” institutions for residents' rotations during the same two evaluation periods.

Residents rotate through all three hospitals during the study periods, and some residents graduated while others started residency during the study periods.

### 2.1. Data Analysis

Statistical analyses on the residents' responses to the survey questions were carried out using linear regression analysis and modeled the mean response of each of the eight survey questions separately. The intervention period was coded using binary “treatment” indicator (0: first 24 months before lecture series and 1: for last 14 months after the lecture series began). The hospital site was modeled as a three-level factor variable (1: Stanford, 2: Valley, and 3: VA). Our regression model allowed for hospital specific profiles across the intervention period. We investigated the effect of the intervention using multivariate Wald test statistic. Additionally, we performed pairwise comparisons for the mean differences in postintervention minus preintervention scores between (a) Stanford and Valley and (b) Stanford and VA. We use robust (sandwich) estimates of variance in our test statistics. For each survey question, two pairwise comparisons were made to investigate differences in the mean postintervention period and preintervention period change scores between the residents' scores at (a) Stanford Medical Center and Valley Hospital and (b) Stanford and VA General. The statistical tests were not adjusted for the two comparisons. All test statistics and associated *p* values are for two-sided tests.

## 3. Results

One hundred and thirty-one residents were eligible to participate in the study. The percentage of residents who completed evaluations was high. The average percentage of completed surveys ranged from 77 percent to 87 percent for the eight questions.

The average number of monthly rotation evaluations completed by the residents was 5 (SD = 3.5) with a minimum of 1 to a maximum of 18 evaluations.  Table 1 displays the estimated means and SD for the performance scores for the preintervention period and postintervention period for each of the three hospitals.

The analyses to evaluate the effectiveness of the intervention are provided in Table 2. For each of the eight questions, we first estimated the mean change in the performance scores between the postintervention period and preintervention period for each hospital and then performed pairwise comparisons of the mean changes scores between Stanford and Valley and Stanford and VA.

**Figure 1:**
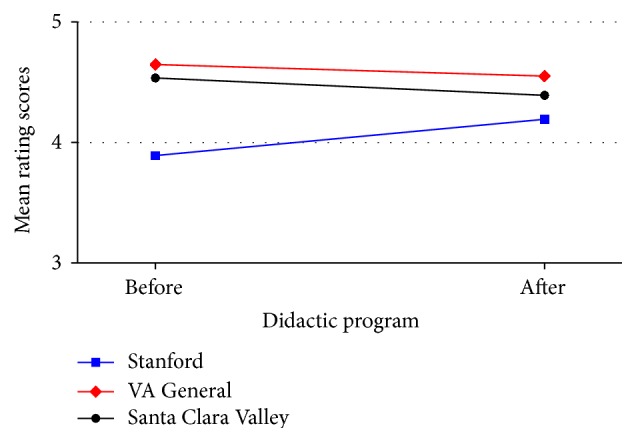
Hospital-level summary statistics for the primary endpoint. Q8: overall teaching quality of this rotation, interaction plot.

The estimates are all positive, indicating that the postintervention less preintervention change estimates for Stanford were all larger than the change estimates for either the Valley or the VA rotations. These differences do not appear to be significant for the scores in Question (1) “the goals of the rotation were defined.” The differences in the change scores appear to be marginal for Question (2) “the goals of the rotation were achieved” and (3) “quality of the syllabus.” For the primary endpoint, Question (8) “overall teaching quality of this rotation,” the comparisons of the mean change scores appear to be significantly different for both (a) Stanford and Valley Hospital and (b) Stanford and VA General (Figure 1). Mean performance scores improved at Stanford, while the mean scores for the Valley and VA rotations declined slightly.

## 4. Discussion

This study attempted to use tools for effective curriculum development including identification of a problem, examining the particular needs of the anesthesia house staff, choosing educational strategies that best fit the intended ABA keyword material, devising steps for implementation, and reviewing program evaluations.

This study provides evidence that a daily 15-minute mini-lecture given by the same faculty member three times each weekday significantly improved resident evaluations of overall teaching quality of a clinical OR rotation compared to controls. On a 5-point Likert-type scale (Unsatisfactory—1, Below Average—2, At Expectation—3, Above Expectation—4, and Outstanding—5) the mean score on “overall teaching quality of this rotation” increased significantly from 3.9 (SD 0.8) to 4.2 (SD 0.7) after addition of the lecture series, whereas the scores decreased slightly at the comparison sites. Compared to both other two hospital rotations, questions items “clinical teaching” and “teaching not directly involved with case management” also improved significantly. Importantly, the development of this consistent, daily discussion of an ABA keyword between residents and an attending was based on resident input to maximize resident acceptance and participation.

The residency has many different one-month rotations that residents rotate. Residents evaluate all the rotations at the end of the month and there is variability in average scores for each of the rotations. For example, for “overall teaching quality of this rotation” some rotations typically score below 4 out of the maximum of 5, while other rotations score near 5. For the Valley and VA general rotations that were used as controls the scores were higher at baseline than the Stanford rotation that implemented the new daily lectures series. The higher scores at the VA General and Valley rotations are likely due to several factors including resident perceptions of the learning environment, the balance between service and education, and the amount of direct teaching by faculty to house staff. The Stanford rotation was chosen for this study as one to improve based on the low baseline scores and the feedback from residents to improve the “education” of the rotation as there was “not enough teaching.”

Applying the principles of learning was crucial for the intervention's success. From an instructional design perspective, well-designed lectures should encourage schema construction [2]. For our intervention, implementing the slide template as a requirement provided a framework for the instruction so the resident could organize the knowledge. Constructed schemas may become automated if they are repeatedly applied and may help leading to desired results.

These design elements are also consistent with cognitive load theory which provides a framework for how people learn. Two channels exist in the human information processing system: one that processes information presented in a visual or pictorial format, such as in projected slides, and the other which processes information presented in an auditory or verbal format, such as in group discussion. In addition, the human cognitive system has a limited working memory that can hold no more than five to nine information elements and can actively process up to two to four elements simultaneously. Visual and auditory working memories are partially independent. The lecture series intervention aims to provide multiple sources of information presented in visual form (e.g., a written text and a diagram), in spoken form, and in group interaction so as not to overload the visual processor.

Also, when house staff find positive value in an activity and perceive support from the environment, they are likely to be motivated to learn. Three important levers—value, efficacy expectancies, and the supportive nature of the environment—were present. Value was appreciated by the residents because annual written exams exist that are required to continue advancing in residency after the second postgraduate year and for board certification after graduation.

The learning environment was supportive due to the engagement of all faculty in these new formal classroom-based teaching sessions. This project's feasibility in a busy clinical environment was facilitated by assuring that the faculty member presenting the day's lecture would be free from clinical duties during lecture times.

Another observed benefit is this format's efficiency. One faculty member teaches three small group sessions per day on a topic on which they have considerable expertise. This format is more efficient than, for example, having 20 attendings give a mini-lecture in 20 different operating rooms to a single resident at various times during the day.

Residents often mentioned the innovative lecture series in the written-in comments of the rotation evaluation. These qualitative comments revealed several themes: (1) short duration: “not requiring a long attention span because the material is presented in bite sized packets” and “It's one of my favorite things I don't think people can learn for more than 15 or 20 minutes at a time, so this is the ideal way to do teaching”; (2) motivation for learning: “great to have learning points at some point of the day that may be completely unrelated to my cases as it helps me to stay more intellectually engaged throughout the day” and “the lecture series is amazing. It keeps me learning and motivates me to read. It's high-yield, focused, and very enjoyable”; (3) exam preparation: “covers topics I might not otherwise study”; “it also has helped me study for tests. Especially if the material covered each session is of manageable scope you remember key things” and “helps me retain facts that are small but important.”; (4) clinical application: “I often revisit lectures relevant to cases I am doing,” “Provide a jumping off point for intraoperative self-directed learning,” and “I definitely preop in a more informed way after the lecture series.”

A recent focus group study of internal medicine residents suggested similar elements as important: awareness of residents' motivation for attending or not attending conference, perception of topics as clinically relevant and readily applicable, desire for shorter sessions, and a safe learning environment [3]. For our lecture series, typically 4–10 residents attended each session at the anesthesia library with learners sitting around a table. This gives the opportunity for small group teaching setting expectations for participation and active learning, as well as providing an additional small break with the possibility of socialization among different classes of residents. Offering identical lectures at 3 different time slots improved the ability to relieve residents to attend.

Using a small group format was better than using a large lecture format in other settings as well [4]. Additionally, engagement in problem-based learning can increase residents' self-directed learning behaviors [5]. For example, the ACTIVE teaching format facilitates small group interaction and requires faculty members to focus on 3–5 learning points centered on cases and questions that allow for discussion [6].

## 5. Limitations

Using the Kirkpatrick model for program evaluation [7], this study focused on the reaction of the learners to evaluate their subjective assessment of the new lecture series. One limitation of this study is that higher Kirkpatrick levels, such as comparing residents test scores to determine actual learning or changes in resident behavior or evaluating how patients were managed, were not assessed.

The study design and data collection posed unique limitations for this study. First, the design was quasi-experimental interrupted time series design. Proponents of the design claim that the design is useful when the effect of the intervention is immediate and pronounced. The design has limitations as most observational study designs. If other events occur in sequence with the intervention and are associated with the outcomes being measured, the resulting statistical inference will be subject to confounding biases. There are other mechanisms that may threaten the internal validity of these results. An additional limitation of the design was that the residents' data were deidentified, making it difficult to account for repeated measures correlation of the residents' responses over time. We were able to determine each eligible resident's rotation location over the 38-month period of the study and performed Monte Carlo simulations for each of the eight questions to investigate the robustness of our results. We generated the mean response profiles at the month-level for each of the three hospitals and using the information of the location information for the residents generated repeated measures correlated responses for very modest correlations (*r* = 0.05) to very strong correlation (*r* = 0.9). We then omitted observations using a missing at random (MAR) mechanism, allowing for the missing values to differ from hospital to hospital and in accordance with the missing data patterns observed for each question. Ten thousand simulation runs were performed for each level of correlation and for each question. We concluded that our observed results, which accounted for variance heterogeneity but could not account for repeated measures correlation, were robust findings. It seems that the robustness of the findings is due to the fact that predictor for the intervention period was neither a purely within-person predictor nor between-person predictor.

Another limitation is that being a single-institution, single-specialty study may limit generalizability because any education intervention has many aspects applicable to the local setting. Although the format may be adapted to fit other residency structures and does not require special technology, an intervention that works well in one department may not necessarily work well in other clinical sites.

## 6. Conclusions

Although the traditional lecture for 45 minutes in a dark conference room remains common in graduate medical education, the new millennial resident learners may require different and innovative instructional methods. This study found that residents evaluations of teaching quality during an anesthesia clinical rotation improve with a lecture series that includes succinct presentations, summaries of keywords in a repetitive standardized slide template, active learning facilitated by reviewing a relevant multiple choice question in a supportive environment, and a focused curriculum on keywords that prepare for in-training written examinations.

## Figures and Tables

**Table 1:** Descriptive summary scores by hospital.

Question	Stanford				Valley				VA General			
	Before		After		Before		After		Before		After	
	Mean	SD	Mean	SD	Mean	SD	Mean	SD	Mean	SD	Mean	SD
(1) The goals of the rotation were defined	3.9	0.9	4.0	0.8	4.4	0.7	4.3	0.8	4.4	0.8	4.4	0.8
(2) The goals of the rotation were achieved	3.9	0.9	4.0	0.8	4.6	0.6	4.3	0.8	4.5	0.7	4.4	0.7
(3) Quality of the syllabus	3.8	0.9	4.0	0.9	4.4	0.6	4.2	0.8	4.4	0.8	4.4	0.8
(4) Were the cases of educational value	4.1	0.8	4.2	0.7	4.7	0.6	4.4	0.8	4.6	0.6	4.4	0.7
(5) Clinical teaching	3.9	0.9	4.2	0.7	4.5	0.7	4.3	0.8	4.7	0.6	4.6	0.6
(6) Teaching not directly involved with case management	3.8	0.9	4.1	0.8	4.3	0.8	3.7	1.0	4.6	0.6	4.4	0.8
(7) Feedback provided	3.6	0.9	4.0	0.9	4.1	0.9	4.0	0.9	4.4	0.8	4.5	0.7
(8) Overall teaching quality of this rotation	3.9	0.8	4.2	0.7	4.5	0.7	4.4	0.8	4.6	0.6	4.6	0.6

**Table 2:** Pairwise comparisons for post-pre estimates.

Question	Pairwise comparison	Est.	SE	*z*-score	*p* value	L95%	U95%
(1) The goals of the rotation were defined	Stanford-Valley	0.31	0.17	1.86	0.063	−0.02	0.64
	Stanford-VA General	0.11	0.17	0.66	0.507	−0.22	0.44

(2) The goals of the rotation were achieved	Stanford-Valley	0.37	0.16	2.26	0.024	0.05	0.69
	Stanford-VA General	0.18	0.16	1.14	0.256	−0.13	0.48

(3) Quality of the syllabus	Stanford-Valley	0.39	0.19	2.10	0.037	0.02	0.76
	Stanford-VA General	0.12	0.18	0.65	0.515	−0.23	0.46

(4) Were the cases of educational value	Stanford-Valley	0.43	0.16	2.73	0.007	0.12	0.73
	Stanford-VA General	0.35	0.14	2.43	0.016	0.07	0.64

(5) Clinical teaching	Stanford-Valley	0.48	0.16	2.91	0.004	0.15	0.80
	Stanford-VA General	0.43	0.14	3.12	0.002	0.16	0.69

(6) Teaching not directly involved with case management	Stanford-Valley	0.90	0.21	4.34	0.000	0.49	1.30
	Stanford-VA General	0.49	0.17	2.97	0.003	0.17	0.82

(7) Feedback provided	Stanford-Valley	0.51	0.19	2.63	0.009	0.13	0.89
	Stanford-VA General	0.23	0.17	1.41	0.160	−0.09	0.56

(8) Overall teaching quality of this rotation	Stanford-Valley	0.45	0.16	2.79	0.005	0.13	0.76
	Stanford-VA General	0.40	0.14	2.87	0.004	0.13	0.67
